# Crystal structure and Hirshfeld surface analysis study of (*E*)-1-(4-chloro­phen­yl)-*N*-(4-ferrocenylphen­yl)methanimine

**DOI:** 10.1107/S2056989021008033

**Published:** 2021-08-10

**Authors:** Riham Sghyar, Oussama Moussaoui, Nada Kheira Sebbar, Younesse Ait Elmachkouri, Ezaddine Irrou, Tuncer Hökelek, Joel T. Mague, Abdesslam Bentama, El Mestafa El hadrami

**Affiliations:** a Laboratory of Applied Organic Chemistry, Sidi Mohamed Ben Abdellah University, Faculty of Sciences and Techniques, Road Immouzer, BP 2202 Fez, Morocco; b Applied Chemistry and Environment Laboratory, Applied Bioorganic Chemistry Team, Faculty of Science, Ibn Zohr University, Agadir, Morocco; c Department of Physics, Hacettepe University, 06800 Beytepe, Ankara, Turkey; d Department of Chemistry, Tulane University, New Orleans, LA 70118, USA

**Keywords:** crystal structure, ferrocen­yl, imine, C—H⋯π(ring) inter­action

## Abstract

The substituted cyclo­penta­dienyl ring in the title mol­ecule, [Fe(C_5_H_5_)(C_18_H_13_ClN)], is nearly coplanar with the phenyl-1-(4-chloro­phen­yl)methanimine substituent, with dihedral angles between the planes of the phenyl­ene ring and the Cp and 4-(chloro­phen­yl)methanimine units of 7.87 (19) and 9.23 (10)°, respectively. The unsubstituted cyclo­penta­dienyl ring is rotationally disordered, the occupancy ratio for the two orientations refined to a 0.666 (7)/0.334 (7) ratio. In the crystal, the mol­ecules pack in ‘bilayers’ parallel to the *ab* plane with the ferrocenyl groups on the outer faces and the substituents directed towards the regions between them. The ferrocenyl groups are linked by C—H⋯π(ring) inter­actions. A Hirshfeld surface analysis of the crystal structure indicates that the most important contributions for the crystal packing are from H⋯H (46.1%), H⋯C/C⋯ H (35.4%) and H⋯Cl/Cl⋯H (13.8%) inter­actions. Thus C—H⋯π(ring) and van der Waals inter­actions are the dominant inter­actions in the crystal packing.

## Abstract 2

The unsubstituted cyclo­penta­dienyl ring is rotationally disordered while the other Cp ring and its substituent are close to coplanar. In the crystal, the mol­ecules pack in ‘bilayers’ parallel to the *ab* plane with the ferrocenyl groups on the outer faces and the substituents directed towards the regions between them. The ferrocenyl groups are linked by C—H⋯π(ring) inter­actions.

## Chemical context   

Compounds containing metallocene building units, and particularly ferrocene derivatives, have been studied extensively both in academic and industrial settings (Santos *et al.*, 2017 ▸; Singh *et al.*, 2019 ▸; Ong & Gasser, 2020 ▸). Owing to a favorable combination of chemical and physical properties, ferrocene derivatives are often biologically active, making them attractive pharmacophores for drug design and useful templates in medicinal chemistry research and therapeutic applications including as anti­oxidant (Bugarinović *et al.*, 2018 ▸; Naz *et al.*, 2020 ▸), anti-inflammatory (Yun Guo *et al.*, 2019), anti­malarial (Peter & Aderibigbe, 2019 ▸; Xiao *et al.*, 2020 ▸), anti­leishmanial (Rauf *et al.*, 2016 ▸), anti­cancer (Wang *et al.*, 2020 ▸; Ismail *et al.*, 2020 ▸), anti­plasmodial (García-Barrantes *et al.*, 2013 ▸), anti­convulsant (Adil *et al.*, 2018 ▸) and anti­microbial (Damljanović *et al.*, 2009 ▸) agents. A wide range of therapeutic activities is also associated with ferrocenyl Schiff bases, which have shown exceptionally high activities against pathogenic microbes (Chohan & Praveen, 2000 ▸; Chohan *et al.* 2001 ▸), and these mol­ecules exhibit potent anti­oxidant and DNA-protecting properties (Li & Liu, 2011 ▸). The potential uses of ferrocenyl Schiff bases also include the synthesis of materials for use in electrochemical sensors (Jo *et al.*, 2007 ▸), non-linear optical materials (Yu *et al.*, 2015 ▸), luminescent systems (Fery-Forgues & Delavaux-Nicot, 2000 ▸), homogeneous catalysis (Gibson *et al.*, 2006 ▸), conducting polymers (Tice *et al.*, 2007 ▸) and organometallic polymers (Xue *et al.*, 2001 ▸). The coordination of a variety of metal centers to produce new complexes of ferrocene-derived Schiff base ligands has been studied for their inter­esting anti­bacterial activities compared to the free ligands (Chohan & Praveen, 2000 ▸). Ferrocenyl liquid crystalline Schiff bases, also known as ferrocenomesogens, present inter­esting magnetic properties such as paramagnetism and control of mol­ecular orientation in magnetic fields (Seshadri *et al.*, 2007 ▸; Onofrei *et al.*, 2012 ▸).
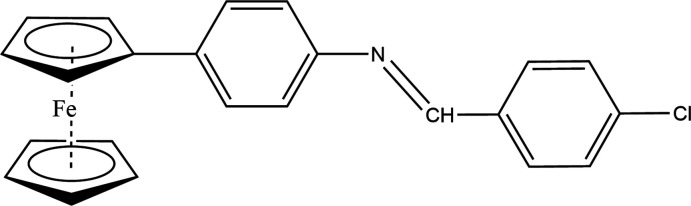


In a continuation of our research towards the synthesis of ferrocene-derived Schiff bases, we have been using 4-ferrocenyl aniline as an inter­mediate in the synthesis of new heterocyclic systems and have studied the condensation reactions between 4-ferrocenyl aniline and 4-chloro­benzaldehyde. The title compound (I) was obtained and characterized by single crystal X-ray diffraction techniques as well as by Hirshfeld surface analysis.

## Structural commentary   

**Figure 1:**
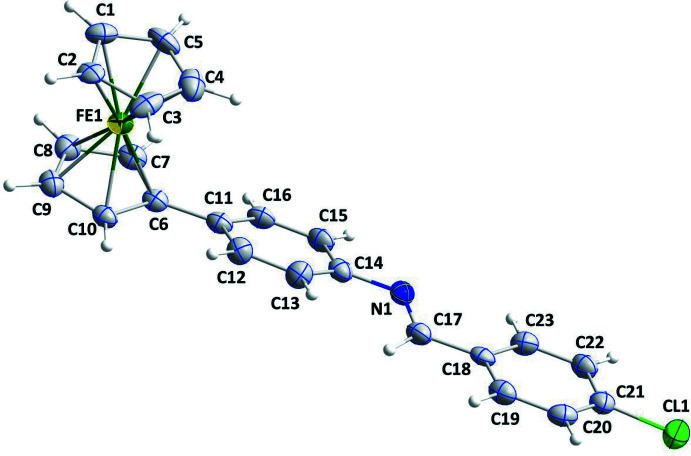
The asymmetric unit of the title compound with the atom-numbering scheme. Displacement ellipsoids are drawn at the 50% probability level. Only the major orientation of the disordered cyclo­penta­dienyl ring is shown.

4-Ferrocenyl aniline was synthesized according to a reported procedure (Hu *et al.*, 2001; Ali *et al.*, 2013) and single crystals of its condensation product with 4-chloro­benzaldehyde were obtained by recrystallization from methanol (Fig. 1 ▸). Bond distances and angles are in the expected ranges and agree well with values observed for similar compounds (see *e.g*. Kumar *et al.*, 2020 ▸; Shabbir *et al.*, 2017 ▸; Toro *et al.*, 2018 ▸). The unsubstituted cyclo­penta­dienyl ring, C1–C5, was found to be rotationally disordered, with a refined occupancy of 0.666 (7) for the major moiety. The two Cp rings are not quite parallel as there is a 2.7 (5)° dihedral angle between them. The substituted cyclo­penta­dienyl ring, C6–C10, is nearly coplanar with the phenyl-1-(4-chloro­phen­yl)methanimine substituent. The Cp ring is inclined by 16.8 (2)° with respect to the C11–C16 phenyl­ene ring. The imine fragment is essentially coplanar with the chloro­phenyl unit, with an r.m.s. deviation from planarity of only 0.05 Å. The dihedral angle between the phenyl­ene ring and the plane of the 4-chloro­phenyl-methanimine unit, N1/C17–C23, is 9.23 (10)°. This renders the entire mol­ecule, with the exception of the Fe atom and the unsubstituted Cp ring, mostly flat.

## Supra­molecular features   

**Figure 2:**
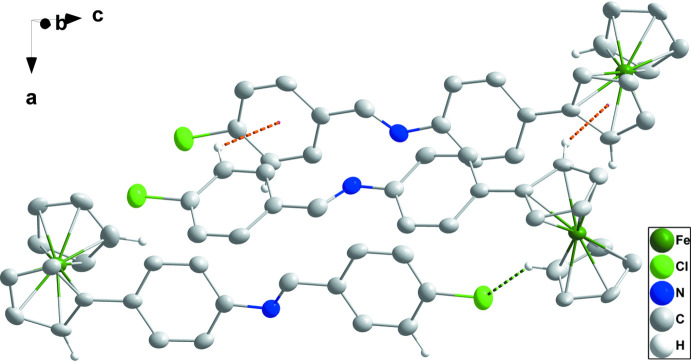
Detail of the inter­molecular inter­actions. C—H⋯Cl hydrogen bonds and C—H⋯π(ring) inter­actions are depicted, respectively, by green and orange dashed lines. Non-inter­acting H atoms are omitted for clarity.

In the crystal, mol­ecules are arranged in double layers perpendicular to the *c* axis with alternating ferrocenyl and Schiff base segments, with the ferrocenyl groups facing towards the outside of each layer and bordering the ferrocene moieties of the neighboring layer, and the phenyl-1-(4-chloro­phen­yl)methanimine substituents at the center of the double layers with the substituents from both sides of the layer inter­digitating with each other (Figs. 2 ▸ and 3 ▸). Two double layers are found within the boundaries of the ortho­rhom­bic *Pbca* unit cell. The phenyl-1-(4-chloro­phen­yl)methanimine substituents are thus all arranged parallel to each other (at the center of each layer). They are, however, rotated along their long axis with respect to each other, and despite their nearly coplanar nature that predestines them for π-stacking inter­actions, no such inter­actions are observed in the solid state. Indeed, directional inter­actions are sparse in the structure of the title compound. Ferrocenyl groups are tied together by C—H⋯π inter­actions, facilitated by neighboring ferrocene units within each layer being roughly 90° rotated against each other. Cp-H atoms thus point towards the π-system of neighboring Cp rings. The shortest C—H⋯π inter­actions are between H5 and H7 towards the C atoms C7 and C10 of the substituted Cp ring at −*x* + 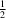, *y* + 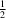, *z* (H⋯C distances are 2.77 and 2.73 Å, respectively), and between H3 and H10 towards C atoms C4 and C3 at −*x* + 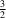, *y* − 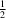, *z* (H⋯C distances are 2.84 and 2.82 Å, respectively). The shortest C—H centroid inter­action is for C7—H7⋯*Cg*2 [*Cg*2 is the centroid of the substituted Cp ring, C6–C10, at −*x* + 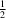, *y* + 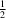, *z*; H⋯*Cg*2 = 2.76 Å, C7⋯*Cg*2 = 3.683 (4) Å, C7—H7⋯*Cg*2 = 154°]. Also present is a C22—H22⋯*Cg*5 inter­action [*Cg*5 is the centroid of the C18–C23 ring at −*x* + 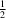, *y* + 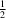, *z* with H⋯*Cg*5 = 2.95 Å, C22⋯*Cg*5 = 3.605 (4) Å, C22—H22⋯*Cg*5 = 127°] and a weak C4—H4⋯Cl1 hydrogen bond (Cl1 at −*x* + 1, *y* + 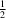, −*z* + 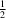, with H4⋯Cl1 = 2.82 Å, C4⋯Cl1 = 3.66 (4) Å and C4—H4⋯Cl1 = 142°).

## Hirshfeld surface analysis   

**Figure 4:**
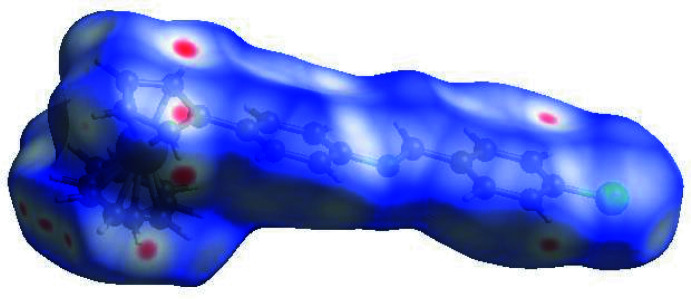
View of the three-dimensional Hirshfeld surface of the title compound, plotted over *d*_norm_ in the range −0.1325 to 1.1632 a.u. The red dots indicate the C—H⋯π(ring) inter­actions involving the ferrocene and the C18–C23 ring.

**Figure 5:**
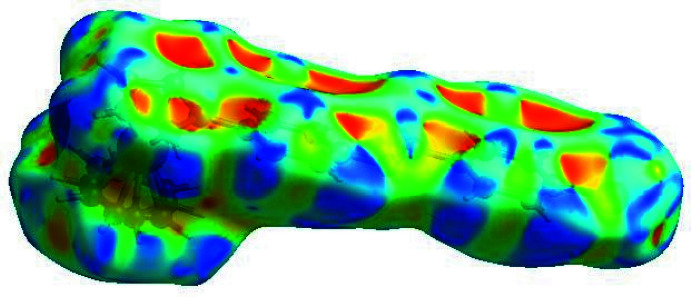
Hirshfeld surface of the title compound plotted over shape-index.

**Figure 6:**
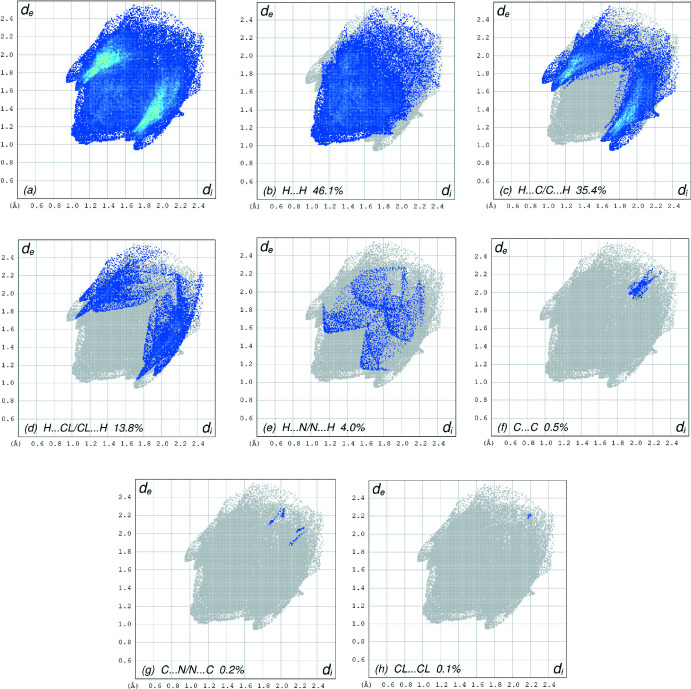
The full two-dimensional fingerprint plots for the title compound, showing (*a*) all inter­actions, and delineated into (*b*) H⋯H, (*c*) H⋯C/C⋯H, (*d*) H⋯Cl/Cl⋯H, (*e*) H⋯N/N⋯H, (*f*) C⋯C, (*g*) C⋯N/N⋯C and (*h*) Cl⋯Cl inter­actions. The *d*_i_ and *d*_e_ values are the closest inter­nal and external distances (in Å) from given points on the Hirshfeld surface.

In order to visualize the inter­molecular inter­actions in the crystal of the title compound, a Hirshfeld surface (HS) analysis (Hirshfeld, 1977 ▸) was carried out using *Crystal Explorer 17.5* (Turner *et al.*, 2017 ▸). In the HS plotted over *d*_norm_ (Fig. 4 ▸), the white surface indicates contacts with distances equal to the sum of van der Waals radii, and the red and blue colors indicate distances shorter (in close contact) or longer (distinct contact) than the van der Waals radii, respectively (Venkatesan *et al.*, 2016 ▸). The bright-red spots indicate their roles as the respective donors and/or acceptors. The blue regions indicate positive electrostatic potentials (hydrogen-bond donors), while the red regions indicate negative electrostatic potentials (hydrogen-bond acceptors). The shape-index of the HS is a tool to visualize π–π stacking by the presence of adjacent red and blue triangles; the absence of adjacent red and/or blue triangles, Fig. 5 ▸, indicates that there are no π–π inter­actions. The overall two-dimensional fingerprint plot is shown in Fig. 6 ▸ *a*, and those delineated into H⋯H, H⋯C/C⋯H, H⋯Cl/Cl⋯H, H⋯N/N⋯H, C⋯C, C⋯N/N⋯C and Cl⋯Cl contacts (McKinnon *et al.*, 2007 ▸) are illustrated in Fig. 6 ▸ *b*–*h*, respectively, together with their relative contributions to the Hirshfeld surface. The most important inter­action is H⋯H, contributing 46.1% to the overall crystal packing, which is reflected in Fig. 6 ▸ *b* as widely scattered points of high density due to the large hydrogen content of the mol­ecule. The presence of C—H⋯π inter­actions, as described in the *Supra­molecular features* section, is indicated by pairs of characteristic wings in the fingerprint plot representing H⋯C/C⋯H contacts, Fig. 6 ▸ *c*. These H⋯C/C⋯H contacts represent a 35.4% contribution to the HS. Pairs of scattered points of spikes are seen in the fingerprint plot delineated into H⋯Cl/Cl⋯H contacts, Fig. 6 ▸ *d*, with a 13.8% contribution to the HS. H⋯N/N⋯H contacts, Fig. 6 ▸ *e*, contribute only 4.0% to the HS. Finally, C⋯C (Fig. 6 ▸ *f*), C⋯N/N⋯C (Fig. 6 ▸ *g*) and Cl⋯Cl contacts (Fig. 6 ▸ *h*) have only 0.5%, 0.2% and 0.1% contributions.

The Hirshfeld surface analysis confirms the importance of H-atom contacts in establishing the packing. The large number of H⋯H and H⋯C/C⋯H inter­actions suggest that C—H⋯π and van der Waals inter­actions play the major role in the crystal packing (Hathwar *et al.*, 2015 ▸).

## Database survey   

**Figure 7:**
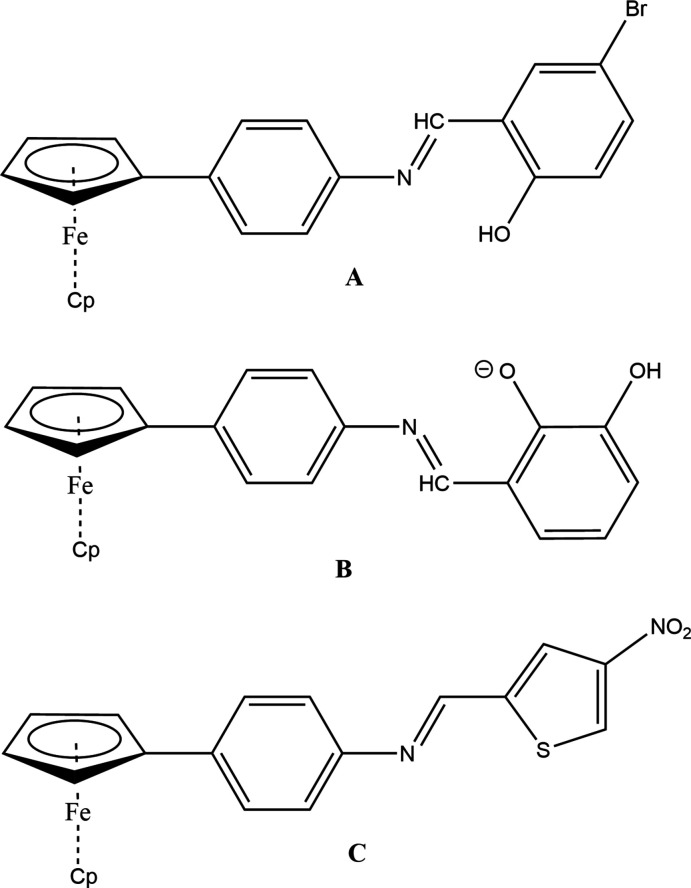
Related ferrocene–Schiff base complexes.

A search of the Cambridge Structural Database (CSD) (Groom *et al.*, 2016, updated to May 29, 2021) found three closely related, ferrocene-substituted Schiff base compounds: (**A**: Jakku *et al.*, 2020 ▸; **B**: Shabbir *et al.*, 2017 ▸; **C**: Toro *et al.*, 2018 ▸; Fig. 7 ▸).

## Synthesis and crystallization   

4-Ferrocenyl aniline was synthesized according to a reported procedure (Hu *et al.*, 2001; Ali *et al.*, 2013). In a 250 mL round-bottom flask, 1.0 mmol of 4-ferrocenyl aniline in 15 mL of dried methanol was mixed with an equimolar amount of 4-chloro­phenyl aldehyde in 15 mL of dried methanol. The mixture was agitated under reflux, the progress of the reaction was monitored by TLC, and the desired product was formed within 6 h. The solvent was removed under vacuum and the solid that was obtained was recrystallized from methanol (yield: 87%) to yield brown crystals, m.p. 210 K. ^1^H NMR (300 MHz, CDCl_3_) δ 4.08 (*s*, 5H, Cp C_5_H_5_); 4.36 (*t*, 2H, Cp C_5_H_4_, *J* = 3.39) ; 4.68 (*t*, 2H, Cp C_5_H_4_, *J* = 3.45); 7.20 (*d*, 2H, C_6_H_4-ar_, *J* = 8.4); 7.48 (*d*, 2H, C_6_H_4-ar_, *J* = 8.43); 7.53 (*d*, 2H, C_6_H_4-ar_, *J* = 8.43); 7.88 (*d*, 2H, C_6_H_4-ar_, *J* = 8.44) ; 8.52 (*s*, 1H, CH=N). ^13^C NMR (75 MHz, CDCl_3_) δ 66.42 (2C, C_5_H_4_); 69.05 (2C, C_5_H_4_); 69,64 (5C, C_5_H_5_); 84.80 (Cq, C_5_H_4_); 121.10 (2C, CH_-Ar_); 126.76 (2C, CH_-Ar_); 129.09 (2C, CH_-Ar_); 129.87 (2C, CH_-Ar_); 134.92 (1Cq, Ar_-CH=N_); 137.20 (1Cq, Ar_-Cl_); 137.72 (1Cq, Ar_-C5H4_); 149.21 (1Cq, Ar_-N=CH_) ; 157.62 (1C, CH=N).

## Refinement   

Crystal, data collection and refinement details are presented in Table 1 ▸. Analysis of 1284 reflections having *I*/σ(*I*) > 15 and chosen from the full data set with *CELL_NOW* (Sheldrick, 2008 ▸) showed the crystal to be either split or non-merohedrally twinned. The top choice of unit cell had parameters *a* = 7.662, *b* = 10.009, *c* = 45.974 Å, *α* = 90.05, *β* = 90.21, *γ* = 89.97° (unrefined) with a second component (14%) rotated 180° about the *b* axis. To eliminate possible bias, the raw data were processed as triclinic using the multi-component version of *SAINT* (Bruker, 2020 ▸) under control of the two-component orientation file generated by *CELL_NOW*, leading to an ortho­rhom­bic cell within experimental error and a twin matrix of: −0.99988 − 0.00291 − 0.00258 − 0.00684 0.99978 0.00453 0.09083 0.09422 − 0.99967, thus indicating presence of two separate domains not related by twinning (‘split crystal’). The data were corrected for absorption using *TWINABS* (Sheldrick, 2009 ▸), which was also used to extract a single-component reflection file from the two-component intensity data, which was used to determine the space group and solve the structure. The resulting space group of *Pbca* required transformation of the original cell by the matrix: 0 1 0 1 0 0 0 0 −1. Trial final refinements with the single-component reflection file and with the complete two-component data showed the former to be more satisfactory on the basis of a lower values for *R*1 and su’s on derived parameters as well as smaller residual features about the Fe atom.

H atoms attached to carbon were placed in calculated positions (C—H = 0.95–1.00 Å). All were included as riding contributions with isotropic displacement parameters 1.2–1.5 times those of the parent atoms. The unsubstituted cyclo­penta­dienyl ring is rotationally disordered over two sets of sites with the two components refined as rigid penta­gons (AFIX 56 constraint of *SHELXL*). ADPs of equivalent major and minor disordered C atoms were constrained to be identical. The occupancy ratio for the two orientations refined to a 0.666 (7)/0.334 (7) ratio.

## Supplementary Material

Crystal structure: contains datablock(s) I, global. DOI: 10.1107/S2056989021008033/zl5016sup1.cif

Structure factors: contains datablock(s) I. DOI: 10.1107/S2056989021008033/zl5016Isup2.hkl

Supporting information file. DOI: 10.1107/S2056989021008033/zl5016Isup3.cdx

CCDC reference: 2101472

Additional supporting information:  crystallographic information; 3D view; checkCIF report

## Figures and Tables

**Figure 3:**
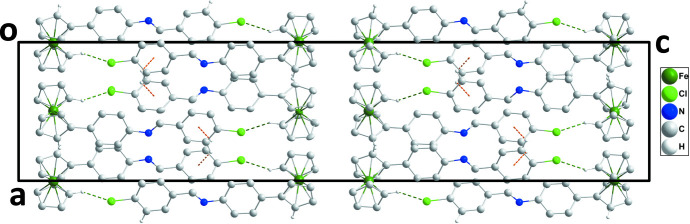
Packing viewed along the *b*-axis direction with inter­molecular inter­actions depicted as in Fig. 2 ▸. Non-inter­acting H atoms are omitted for clarity.

**Table 1:** Experimental details

Crystal data	
Chemical formula	[Fe(C_5_H_5_)(C_18_H_13_ClN)]
*M*_r_	399.68
Crystal system, space group	Orthorhombic, *P* *b* *c* *a*
Temperature (K)	150
*a*, *b*, *c* (Å)	10.0991 (18), 7.7277 (14), 45.979 (8)
*V* (Å^3^)	3588.3 (11)
*Z*	8
Radiation type	Mo *K*α
μ (mm^−1^)	1.00
Crystal size (mm)	0.13 × 0.12 × 0.04

Data collection	
Diffractometer	Bruker D8 QUEST PHOTON 3 diffractometer
Absorption correction	Multi-scan (*TWINABS*; Sheldrick, 2009 ▸)
*T*_min_, *T*_max_	0.88, 0.96
No. of measured, independent and observed [*I* > 2σ(*I*)] reflections	12370, 4001, 2903
*R*_int_	0.046
(sin θ/λ)_max_ (Å^−1^)	0.653

Refinement	
*R*[*F*^2^ > 2σ(*F* ^2^)], *wR*(*F* ^2^), *S*	0.063, 0.118, 1.17
No. of reflections	4001
No. of parameters	233
H-atom treatment	H-atom parameters constrained
Δρ_max_, Δρ_min_ (e Å^−3^)	0.44, −0.38

Computer programs: *APEX3* and *SAINT* (Bruker, 2020 ▸), *SHELXT* (Sheldrick, 2015*a* ▸), *SHELXL2018/1* (Sheldrick, 2015*b* ▸), *DIAMOND* (Brandenburg & Putz, 2012 ▸) and *SHELXTL* (Sheldrick, 2008 ▸).

## supplementary crystallographic information

### Crystal data

**Table:**

[Fe(C_5_H_5_)(C_18_H_13_ClN)]	*D*_x_ = 1.480 Mg m^−^^3^
*M**_r_* = 399.68	Mo *K*α radiation, λ = 0.71073 Å
Orthorhombic, *P**b**c**a*	Cell parameters from 7602 reflections
*a* = 10.0991 (18) Å	θ = 2.7–27.6°
*b* = 7.7277 (14) Å	µ = 1.00 mm^−^^1^
*c* = 45.979 (8) Å	*T* = 150 K
*V* = 3588.3 (11) Å^3^	Plate, orange
*Z* = 8	0.13 × 0.12 × 0.04 mm
*F*(000) = 1648	

### Data collection

**Table:**

Bruker D8 QUEST PHOTON 3 diffractometer	4001 independent reflections
Radiation source: fine-focus sealed tube	2903 reflections with *I* > 2σ(*I*)
Graphite monochromator	*R*_int_ = 0.046
Detector resolution: 7.3910 pixels mm^-1^	θ_max_ = 27.6°, θ_min_ = 1.8°
ω scans	*h* = −13→12
Absorption correction: multi-scan (*TWINABS*; Sheldrick, 2009)	*k* = −10→9
*T*_min_ = 0.88, *T*_max_ = 0.96	*l* = −59→0
12370 measured reflections	

### Refinement

**Table:**

Refinement on *F*^2^	Primary atom site location: dual
Least-squares matrix: full	Secondary atom site location: difference Fourier map
*R*[*F*^2^ > 2σ(*F*^2^)] = 0.063	Hydrogen site location: inferred from neighbouring sites
*wR*(*F*^2^) = 0.118	H-atom parameters constrained
*S* = 1.17	*w* = 1/[σ^2^(*F*_o_^2^) + (0.0177*P*)^2^ + 8.9667*P*] where *P* = (*F*_o_^2^ + 2*F*_c_^2^)/3
4001 reflections	(Δ/σ)_max_ = 0.001
233 parameters	Δρ_max_ = 0.44 e Å^−^^3^
0 restraints	Δρ_min_ = −0.38 e Å^−^^3^

### Special details

**Table:**

Experimental. The diffraction data were obtained from 7 sets of frames, each of width 0.5° in ω, collected with scan parameters determined by the "strategy" routine in *APEX3*. The scan time was 40 sec/frame. Analysis of 1284 reflections having I/σ(I) > 15 and chosen from the full data set with *CELL_NOW* (Sheldrick, 2008) showed the crystal to non-merohedrally twinned. The top choice of unit cell had parameters *a* = 7.662, *b* = 10.009, *c* = 45.974 Å, *α* = 90.05, *β* = 90.21, *γ* = 89.97° (unrefined) with a second component (14%) rotated 180° about the *b*-axis. To eliminate possible bias, the raw data were processed as triclinic using the multi-component version of *SAINT* (Bruker, 2020) under control of the two-component orientation file generated by *CELL_NOW* leading to an orthorhombic cell within experinental error and a twin matrix of: -0.99988 -0.00291 -0.00258 -0.00684 0.99978 0.00453 0.09083 0.09422 -0.99967.
Geometry. All esds (except the esd in the dihedral angle between two l.s. planes) are estimated using the full covariance matrix. The cell esds are taken into account individually in the estimation of esds in distances, angles and torsion angles; correlations between esds in cell parameters are only used when they are defined by crystal symmetry. An approximate (isotropic) treatment of cell esds is used for estimating esds involving l.s. planes.
Refinement. Refinement of F^2^ against ALL reflections. The weighted R-factor wR and goodness of fit S are based on F^2^, conventional R-factors R are based on F, with F set to zero for negative F^2^. The threshold expression of F^2^ > 2sigma(F^2^) is used only for calculating R-factors(gt) etc. and is not relevant to the choice of reflections for refinement. R-factors based on F^2^ are statistically about twice as large as those based on F, and R- factors based on ALL data will be even larger. H-atoms attached to carbon were placed in calculated positions (C—H = 0.95 - 1.00 Å). All were included as riding contributions with isotropic displacement parameters 1.2 - 1.5 times those of the attached atoms. The C1···C5 ring is rotationally disordered over two orientations in a 0.666 (7)/0.334 (7) ratio. The two components were refined as rigid pentagons. Trial refinements with the single-component reflection file extracted from the full data set with *TWINABS* and with the complete two-component data showed the former to be more satisfactory on the basis of a lower values for R1 and su's on derived parameters as well as smaller residual features abot the Fe atom.

### Fractional atomic coordinates and isotropic or equivalent isotropic displacement parameters (Å^2^)

**Table:**

	*x*	*y*	*z*	*U*_iso_*/*U*_eq_	Occ. (<1)
Fe1	0.50398 (5)	0.66068 (7)	0.44481 (2)	0.02709 (15)	
Cl1	0.35163 (12)	0.58417 (15)	0.14716 (2)	0.0460 (3)	
N1	0.3450 (3)	0.5987 (4)	0.29393 (6)	0.0302 (7)	
C1	0.5820 (7)	0.8567 (9)	0.46980 (8)	0.0357 (19)	0.666 (7)
H1	0.552750	0.889508	0.489834	0.043*	0.666 (7)
C2	0.6831 (5)	0.7360 (6)	0.46279 (12)	0.0310 (15)	0.666 (7)
H2	0.736892	0.667977	0.477030	0.037*	0.666 (7)
C3	0.6915 (5)	0.7248 (7)	0.43202 (13)	0.039 (2)	0.666 (7)
H3	0.753532	0.649277	0.420787	0.047*	0.666 (7)
C4	0.5956 (7)	0.8385 (9)	0.42001 (7)	0.046 (2)	0.666 (7)
H4	0.579632	0.859268	0.398829	0.056*	0.666 (7)
C5	0.5279 (6)	0.9201 (8)	0.44336 (14)	0.040 (2)	0.666 (7)
H5	0.455543	1.007747	0.441501	0.048*	0.666 (7)
C1A	0.6195 (16)	0.814 (2)	0.46947 (16)	0.0357 (19)	0.334 (7)
H1A	0.633804	0.802489	0.490908	0.043*	0.334 (7)
C2A	0.6952 (9)	0.7317 (14)	0.4474 (3)	0.0310 (15)	0.334 (7)
H2A	0.772102	0.652440	0.450570	0.037*	0.334 (7)
C3A	0.6450 (12)	0.7860 (18)	0.42003 (19)	0.039 (2)	0.334 (7)
H3A	0.678096	0.748136	0.400551	0.047*	0.334 (7)
C4A	0.5383 (11)	0.9017 (17)	0.4252 (3)	0.046 (2)	0.334 (7)
H4A	0.481684	0.957304	0.409988	0.056*	0.334 (7)
C5A	0.5226 (14)	0.9189 (19)	0.4557 (3)	0.040 (2)	0.334 (7)
H5A	0.454299	0.990947	0.465827	0.048*	0.334 (7)
C6	0.3865 (3)	0.5206 (5)	0.41718 (7)	0.0258 (8)	
C7	0.3080 (4)	0.6077 (5)	0.43866 (7)	0.0297 (9)	
H7	0.236759	0.694502	0.434743	0.036*	
C8	0.3495 (4)	0.5481 (5)	0.46672 (8)	0.0339 (9)	
H8	0.312783	0.587107	0.485821	0.041*	
C9	0.4530 (4)	0.4280 (5)	0.46280 (8)	0.0319 (9)	
H9	0.502053	0.367060	0.478674	0.038*	
C10	0.4771 (4)	0.4098 (5)	0.43228 (7)	0.0279 (8)	
H10	0.545311	0.333379	0.423122	0.034*	
C11	0.3788 (4)	0.5450 (5)	0.38538 (7)	0.0271 (8)	
C12	0.4825 (4)	0.4907 (5)	0.36759 (8)	0.0309 (9)	
H12	0.559850	0.442818	0.376174	0.037*	
C13	0.4749 (4)	0.5055 (5)	0.33747 (8)	0.0326 (9)	
H13	0.546303	0.466509	0.325738	0.039*	
C14	0.3630 (4)	0.5770 (5)	0.32443 (7)	0.0290 (8)	
C15	0.2619 (4)	0.6370 (5)	0.34228 (7)	0.0309 (9)	
H15	0.186873	0.691251	0.333770	0.037*	
C16	0.2682 (4)	0.6192 (5)	0.37221 (7)	0.0300 (9)	
H16	0.196511	0.658035	0.383888	0.036*	
C17	0.4136 (4)	0.5112 (5)	0.27598 (8)	0.0318 (9)	
H17	0.475449	0.428943	0.283162	0.038*	
C18	0.3996 (4)	0.5342 (5)	0.24447 (7)	0.0267 (8)	
C19	0.4920 (4)	0.4611 (5)	0.22554 (8)	0.0318 (9)	
H19	0.564591	0.397826	0.233249	0.038*	
C20	0.4793 (4)	0.4793 (5)	0.19553 (8)	0.0330 (9)	
H20	0.543157	0.430314	0.182773	0.040*	
C21	0.3729 (4)	0.5694 (5)	0.18472 (8)	0.0321 (9)	
C22	0.2802 (4)	0.6460 (5)	0.20288 (8)	0.0323 (9)	
H22	0.208362	0.709972	0.195003	0.039*	
C23	0.2942 (4)	0.6279 (5)	0.23271 (8)	0.0316 (9)	
H23	0.231255	0.679942	0.245323	0.038*	

### Atomic displacement parameters (Å^2^)

**Table:**

	*U*^11^	*U*^22^	*U*^33^	*U*^12^	*U*^13^	*U*^23^
Fe1	0.0214 (3)	0.0270 (3)	0.0329 (2)	−0.0033 (3)	0.0021 (2)	−0.0016 (2)
Cl1	0.0552 (7)	0.0485 (7)	0.0341 (5)	−0.0034 (6)	−0.0029 (5)	0.0010 (5)
N1	0.0298 (18)	0.0277 (18)	0.0332 (15)	−0.0038 (16)	−0.0029 (13)	0.0023 (13)
C1	0.034 (5)	0.028 (5)	0.045 (2)	−0.006 (4)	0.005 (2)	−0.011 (2)
C2	0.027 (3)	0.027 (3)	0.039 (4)	−0.010 (2)	−0.012 (3)	−0.003 (3)
C3	0.025 (4)	0.053 (5)	0.038 (4)	−0.017 (3)	0.004 (3)	−0.015 (3)
C4	0.045 (6)	0.052 (6)	0.042 (3)	−0.017 (5)	−0.005 (3)	0.012 (3)
C5	0.031 (3)	0.021 (3)	0.067 (7)	0.000 (2)	−0.005 (4)	0.010 (4)
C1A	0.034 (5)	0.028 (5)	0.045 (2)	−0.006 (4)	0.005 (2)	−0.011 (2)
C2A	0.027 (3)	0.027 (3)	0.039 (4)	−0.010 (2)	−0.012 (3)	−0.003 (3)
C3A	0.025 (4)	0.053 (5)	0.038 (4)	−0.017 (3)	0.004 (3)	−0.015 (3)
C4A	0.045 (6)	0.052 (6)	0.042 (3)	−0.017 (5)	−0.005 (3)	0.012 (3)
C5A	0.031 (3)	0.021 (3)	0.067 (7)	0.000 (2)	−0.005 (4)	0.010 (4)
C6	0.0174 (19)	0.026 (2)	0.0341 (18)	−0.0029 (16)	−0.0007 (14)	0.0006 (15)
C7	0.0172 (19)	0.030 (2)	0.0419 (19)	−0.0017 (17)	0.0034 (15)	0.0000 (16)
C8	0.030 (2)	0.038 (3)	0.0332 (18)	−0.010 (2)	0.0048 (16)	−0.0002 (16)
C9	0.027 (2)	0.034 (2)	0.0351 (18)	−0.0068 (19)	−0.0032 (15)	0.0058 (16)
C10	0.026 (2)	0.023 (2)	0.0356 (17)	−0.0020 (17)	−0.0034 (15)	0.0010 (15)
C11	0.021 (2)	0.023 (2)	0.0373 (18)	−0.0019 (17)	−0.0010 (15)	−0.0002 (15)
C12	0.022 (2)	0.035 (2)	0.0357 (18)	0.0002 (18)	−0.0021 (15)	0.0020 (16)
C13	0.027 (2)	0.037 (2)	0.0336 (18)	0.0003 (19)	0.0021 (15)	−0.0006 (16)
C14	0.029 (2)	0.023 (2)	0.0351 (18)	−0.0041 (18)	−0.0024 (16)	0.0036 (15)
C15	0.026 (2)	0.025 (2)	0.0412 (18)	0.0032 (19)	−0.0073 (16)	0.0014 (16)
C16	0.027 (2)	0.023 (2)	0.0396 (18)	0.0026 (18)	0.0009 (16)	−0.0014 (15)
C17	0.033 (2)	0.023 (2)	0.0394 (19)	0.0002 (19)	−0.0063 (17)	0.0014 (16)
C18	0.028 (2)	0.016 (2)	0.0364 (18)	−0.0043 (17)	−0.0019 (15)	−0.0008 (14)
C19	0.024 (2)	0.027 (2)	0.045 (2)	0.0016 (19)	−0.0066 (17)	0.0003 (16)
C20	0.030 (2)	0.027 (2)	0.0421 (19)	−0.0035 (19)	0.0040 (17)	−0.0049 (16)
C21	0.036 (2)	0.027 (2)	0.0339 (18)	−0.0058 (19)	−0.0020 (16)	0.0015 (16)
C22	0.029 (2)	0.027 (2)	0.0405 (18)	0.0014 (19)	−0.0068 (16)	0.0019 (17)
C23	0.029 (2)	0.028 (2)	0.0375 (18)	0.0003 (18)	−0.0007 (15)	−0.0031 (16)

### Geometric parameters (Å, º)

**Table:**

Fe1—C2A	2.011 (11)	C5A—H5A	1.0000
Fe1—C4	2.011 (5)	C6—C10	1.433 (5)
Fe1—C1A	2.012 (16)	C6—C7	1.434 (5)
Fe1—C5	2.020 (6)	C6—C11	1.476 (5)
Fe1—C7	2.041 (4)	C7—C8	1.432 (5)
Fe1—C10	2.041 (4)	C7—H7	1.0000
Fe1—C3	2.044 (5)	C8—C9	1.409 (6)
Fe1—C9	2.045 (4)	C8—H8	1.0000
Fe1—C6	2.048 (4)	C9—C10	1.431 (5)
Fe1—C8	2.051 (4)	C9—H9	1.0000
Fe1—C1	2.058 (6)	C10—H10	1.0000
Fe1—C3A	2.065 (11)	C11—C16	1.394 (5)
Cl1—C21	1.744 (4)	C11—C12	1.394 (5)
N1—C17	1.272 (5)	C12—C13	1.391 (5)
N1—C14	1.424 (4)	C12—H12	0.9500
C1—C5	1.4200	C13—C14	1.393 (5)
C1—C2	1.4200	C13—H13	0.9500
C1—H1	1.0000	C14—C15	1.390 (5)
C2—C3	1.4200	C15—C16	1.385 (5)
C2—H2	1.0000	C15—H15	0.9500
C3—C4	1.4200	C16—H16	0.9500
C3—H3	1.0000	C17—C18	1.467 (5)
C4—C5	1.4200	C17—H17	0.9500
C4—H4	1.0000	C18—C19	1.395 (5)
C5—H5	1.0000	C18—C23	1.396 (5)
C1A—C2A	1.4200	C19—C20	1.393 (5)
C1A—C5A	1.4200	C19—H19	0.9500
C1A—H1A	1.0000	C20—C21	1.374 (5)
C2A—C3A	1.4200	C20—H20	0.9500
C2A—H2A	1.0000	C21—C22	1.387 (5)
C3A—C4A	1.4200	C22—C23	1.386 (5)
C3A—H3A	1.0000	C22—H22	0.9500
C4A—C5A	1.4200	C23—H23	0.9500
C4A—H4A	1.0000		
			
C2A—Fe1—C1A	41.3 (2)	C1A—C2A—Fe1	69.4 (6)
C4—Fe1—C5	41.25 (11)	C3A—C2A—Fe1	71.7 (5)
C2A—Fe1—C7	173.8 (4)	C1A—C2A—H2A	126.0
C4—Fe1—C7	120.3 (2)	C3A—C2A—H2A	126.0
C1A—Fe1—C7	139.3 (4)	Fe1—C2A—H2A	126.0
C5—Fe1—C7	108.08 (19)	C4A—C3A—C2A	108.0
C2A—Fe1—C10	113.8 (3)	C4A—C3A—Fe1	71.3 (5)
C4—Fe1—C10	123.4 (2)	C2A—C3A—Fe1	67.6 (5)
C1A—Fe1—C10	142.7 (4)	C4A—C3A—H3A	126.0
C5—Fe1—C10	161.7 (2)	C2A—C3A—H3A	126.0
C7—Fe1—C10	68.99 (16)	Fe1—C3A—H3A	126.0
C4—Fe1—C3	40.99 (9)	C5A—C4A—C3A	108.0
C5—Fe1—C3	68.85 (13)	C5A—C4A—Fe1	68.9 (6)
C7—Fe1—C3	155.1 (2)	C3A—C4A—Fe1	68.8 (5)
C10—Fe1—C3	105.79 (16)	C5A—C4A—H4A	126.0
C2A—Fe1—C9	117.2 (3)	C3A—C4A—H4A	126.0
C4—Fe1—C9	161.1 (2)	Fe1—C4A—H4A	126.0
C1A—Fe1—C9	115.8 (3)	C4A—C5A—C1A	108.0
C5—Fe1—C9	156.3 (2)	C4A—C5A—Fe1	71.3 (5)
C7—Fe1—C9	68.63 (16)	C1A—C5A—Fe1	67.6 (5)
C10—Fe1—C9	41.01 (14)	C4A—C5A—H5A	126.0
C3—Fe1—C9	124.3 (2)	C1A—C5A—H5A	126.0
C2A—Fe1—C6	137.5 (4)	Fe1—C5A—H5A	126.0
C4—Fe1—C6	106.01 (16)	C10—C6—C7	107.4 (3)
C1A—Fe1—C6	175.3 (4)	C10—C6—C11	126.2 (3)
C5—Fe1—C6	124.97 (19)	C7—C6—C11	126.4 (3)
C7—Fe1—C6	41.08 (14)	C10—C6—Fe1	69.2 (2)
C10—Fe1—C6	41.03 (14)	C7—C6—Fe1	69.2 (2)
C3—Fe1—C6	119.10 (17)	C11—C6—Fe1	125.3 (3)
C9—Fe1—C6	68.92 (15)	C8—C7—C6	107.9 (3)
C2A—Fe1—C8	144.8 (4)	C8—C7—Fe1	69.9 (2)
C4—Fe1—C8	156.7 (2)	C6—C7—Fe1	69.7 (2)
C1A—Fe1—C8	114.5 (4)	C8—C7—H7	126.0
C5—Fe1—C8	121.9 (2)	C6—C7—H7	126.0
C7—Fe1—C8	40.99 (14)	Fe1—C7—H7	126.0
C10—Fe1—C8	68.56 (16)	C9—C8—C7	108.3 (3)
C3—Fe1—C8	161.6 (2)	C9—C8—Fe1	69.7 (2)
C9—Fe1—C8	40.25 (16)	C7—C8—Fe1	69.1 (2)
C6—Fe1—C8	68.88 (14)	C9—C8—H8	125.8
C4—Fe1—C1	68.74 (15)	C7—C8—H8	125.8
C5—Fe1—C1	40.74 (12)	Fe1—C8—H8	125.8
C7—Fe1—C1	126.60 (19)	C8—C9—C10	108.4 (3)
C10—Fe1—C1	155.2 (2)	C8—C9—Fe1	70.1 (2)
C3—Fe1—C1	68.13 (11)	C10—C9—Fe1	69.3 (2)
C9—Fe1—C1	121.19 (19)	C8—C9—H9	125.8
C6—Fe1—C1	163.2 (2)	C10—C9—H9	125.8
C8—Fe1—C1	109.23 (18)	Fe1—C9—H9	125.8
C2A—Fe1—C3A	40.75 (18)	C9—C10—C6	107.9 (3)
C1A—Fe1—C3A	68.6 (3)	C9—C10—Fe1	69.7 (2)
C7—Fe1—C3A	133.3 (4)	C6—C10—Fe1	69.7 (2)
C10—Fe1—C3A	112.4 (3)	C9—C10—H10	126.0
C9—Fe1—C3A	144.0 (4)	C6—C10—H10	126.0
C6—Fe1—C3A	107.8 (3)	Fe1—C10—H10	126.0
C8—Fe1—C3A	174.1 (4)	C16—C11—C12	118.1 (3)
C17—N1—C14	120.4 (3)	C16—C11—C6	121.7 (3)
C5—C1—C2	108.0	C12—C11—C6	120.2 (3)
C5—C1—Fe1	68.20 (19)	C13—C12—C11	121.2 (4)
C2—C1—Fe1	70.44 (19)	C13—C12—H12	119.4
C5—C1—H1	126.0	C11—C12—H12	119.4
C2—C1—H1	126.0	C12—C13—C14	120.4 (4)
Fe1—C1—H1	126.0	C12—C13—H13	119.8
C1—C2—C3	108.0	C14—C13—H13	119.8
C1—C2—Fe1	69.3 (2)	C15—C14—C13	118.3 (3)
C3—C2—Fe1	68.7 (2)	C15—C14—N1	116.6 (3)
C1—C2—H2	126.0	C13—C14—N1	125.1 (3)
C3—C2—H2	126.0	C16—C15—C14	121.3 (4)
Fe1—C2—H2	126.0	C16—C15—H15	119.3
C4—C3—C2	108.0	C14—C15—H15	119.3
C4—C3—Fe1	68.3 (2)	C15—C16—C11	120.7 (4)
C2—C3—Fe1	70.9 (2)	C15—C16—H16	119.7
C4—C3—H3	126.0	C11—C16—H16	119.7
C2—C3—H3	126.0	N1—C17—C18	121.6 (4)
Fe1—C3—H3	126.0	N1—C17—H17	119.2
C3—C4—C5	108.0	C18—C17—H17	119.2
C3—C4—Fe1	70.74 (19)	C19—C18—C23	118.6 (3)
C5—C4—Fe1	69.7 (2)	C19—C18—C17	120.2 (3)
C3—C4—H4	126.0	C23—C18—C17	121.3 (3)
C5—C4—H4	126.0	C20—C19—C18	121.1 (4)
Fe1—C4—H4	126.0	C20—C19—H19	119.5
C1—C5—C4	108.0	C18—C19—H19	119.5
C1—C5—Fe1	71.1 (2)	C21—C20—C19	118.8 (4)
C4—C5—Fe1	69.0 (2)	C21—C20—H20	120.6
C1—C5—H5	126.0	C19—C20—H20	120.6
C4—C5—H5	126.0	C20—C21—C22	121.8 (3)
Fe1—C5—H5	126.0	C20—C21—Cl1	119.2 (3)
C2A—C1A—C5A	108.0	C22—C21—Cl1	119.0 (3)
C2A—C1A—Fe1	69.3 (5)	C23—C22—C21	119.0 (4)
C5A—C1A—Fe1	71.7 (4)	C23—C22—H22	120.5
C2A—C1A—H1A	126.0	C21—C22—H22	120.5
C5A—C1A—H1A	126.0	C22—C23—C18	120.9 (4)
Fe1—C1A—H1A	126.0	C22—C23—H23	119.6
C1A—C2A—C3A	108.0	C18—C23—H23	119.6
			
C5—C1—C2—C3	0.0	C7—C8—C9—Fe1	58.5 (3)
Fe1—C1—C2—C3	58.00 (19)	C8—C9—C10—C6	−0.1 (4)
C5—C1—C2—Fe1	−58.00 (19)	Fe1—C9—C10—C6	−59.5 (3)
C1—C2—C3—C4	0.0	C8—C9—C10—Fe1	59.4 (3)
Fe1—C2—C3—C4	58.4 (2)	C7—C6—C10—C9	0.6 (4)
C1—C2—C3—Fe1	−58.4 (2)	C11—C6—C10—C9	178.6 (4)
C2—C3—C4—C5	0.0	Fe1—C6—C10—C9	59.4 (3)
Fe1—C3—C4—C5	60.0 (2)	C7—C6—C10—Fe1	−58.8 (3)
C2—C3—C4—Fe1	−60.0 (2)	C11—C6—C10—Fe1	119.2 (4)
C2—C1—C5—C4	0.0	C10—C6—C11—C16	163.4 (4)
Fe1—C1—C5—C4	−59.4 (2)	C7—C6—C11—C16	−18.9 (6)
C2—C1—C5—Fe1	59.4 (2)	Fe1—C6—C11—C16	−107.8 (4)
C3—C4—C5—C1	0.0	C10—C6—C11—C12	−15.5 (6)
Fe1—C4—C5—C1	60.66 (17)	C7—C6—C11—C12	162.2 (4)
C3—C4—C5—Fe1	−60.66 (17)	Fe1—C6—C11—C12	73.3 (5)
C5A—C1A—C2A—C3A	0.0	C16—C11—C12—C13	−1.8 (6)
Fe1—C1A—C2A—C3A	61.6 (4)	C6—C11—C12—C13	177.1 (4)
C5A—C1A—C2A—Fe1	−61.6 (4)	C11—C12—C13—C14	0.7 (6)
C1A—C2A—C3A—C4A	0.0	C12—C13—C14—C15	1.7 (6)
Fe1—C2A—C3A—C4A	60.1 (5)	C12—C13—C14—N1	179.8 (4)
C1A—C2A—C3A—Fe1	−60.1 (5)	C17—N1—C14—C15	−162.1 (4)
C2A—C3A—C4A—C5A	0.0	C17—N1—C14—C13	19.8 (6)
Fe1—C3A—C4A—C5A	57.8 (6)	C13—C14—C15—C16	−3.1 (6)
C2A—C3A—C4A—Fe1	−57.8 (6)	N1—C14—C15—C16	178.7 (3)
C3A—C4A—C5A—C1A	0.0	C14—C15—C16—C11	2.0 (6)
Fe1—C4A—C5A—C1A	57.7 (4)	C12—C11—C16—C15	0.5 (6)
C3A—C4A—C5A—Fe1	−57.7 (4)	C6—C11—C16—C15	−178.4 (4)
C2A—C1A—C5A—C4A	0.0	C14—N1—C17—C18	−178.4 (3)
Fe1—C1A—C5A—C4A	−60.1 (5)	N1—C17—C18—C19	168.2 (4)
C2A—C1A—C5A—Fe1	60.1 (5)	N1—C17—C18—C23	−12.3 (6)
C10—C6—C7—C8	−0.8 (4)	C23—C18—C19—C20	−0.5 (6)
C11—C6—C7—C8	−178.9 (4)	C17—C18—C19—C20	179.0 (4)
Fe1—C6—C7—C8	−59.7 (3)	C18—C19—C20—C21	−0.8 (6)
C10—C6—C7—Fe1	58.9 (3)	C19—C20—C21—C22	1.8 (6)
C11—C6—C7—Fe1	−119.2 (4)	C19—C20—C21—Cl1	−176.9 (3)
C6—C7—C8—C9	0.8 (4)	C20—C21—C22—C23	−1.4 (6)
Fe1—C7—C8—C9	−58.8 (3)	Cl1—C21—C22—C23	177.3 (3)
C6—C7—C8—Fe1	59.6 (3)	C21—C22—C23—C18	0.0 (6)
C7—C8—C9—C10	−0.4 (4)	C19—C18—C23—C22	0.9 (6)
Fe1—C8—C9—C10	−58.9 (3)	C17—C18—C23—C22	−178.7 (4)
